# A Case of Intestinal Injury—Recovery

**Published:** 1892-08

**Authors:** F. C. Johnston

**Affiliations:** Richland, Ga.


### A CASE OF INTESTINAL INJURY—RECOVERY.

BY
F. C. JOHNSTON, M. D.,
RICHLAND, GA.

On the night of the 8th of June, this year, I successfully treated the most severe case of intestinal injury I ever saw, at a station on the railroad above here, seven miles distant. Burl Brown (col.), aged thirty years, while in a fight with another negro received a stab with a six-inch-blade knife in the umbilical region. The blade entered two inches to the left, and one inch below the umbilicus and cut upwards. The wound measured four inches in length and there was complete intestinal evisceration. He remained where he fell one hour with his intestines on the ground. He was afterwards removed to an empty box car where I found him. The hemorrhage had been profuse and there was some collapse. It was impossible to get the help of a brother physician, or any light except one railroad lantern. He was simply lying on the car floor. I did not have a chair or a box to sit on; I had to work on my knees. I will state that the negro was a stranger in the vicinity, and none of the rest of the darkies would let him go to their houses. On examination I found his intestines cut in five separate places, with a two inch cut in the mesentery which required the ligation of two arteries. Fecal matter was escaping from all the gut wounds. There was one gut cut through and through over two inches in length in each wall, and there was not a single wound of the gut that would not have measured over an inch. A bystander administered the chloroform. After thoroughly washing the intestines in warm carbolic acid water (which had previously boiled) I brought the edges of the gut together, and with a round, straight needle, No. 9, threaded with fine silk thread which had been soaking in full strength zymocide, I stitched up all the intestinal wounds, five in number, and the mesentery with a double row of stitches. The intestines were now pulled to one side, and a number of blood clots and a handful of fecal matter was washed out of the abdominal cavity. The intestines were rinsed and returned to their home, and the abdominal wound closed except a place that would admit of the index finger at the lower edge of the wound for drainage. A little iodoform was sprinkled over it and a bandage put around his waist and over the wound. It was eight o'clock P. M. when I began to work, and at ten I left him. I gave him opium in one-fourth grain doses three times per day, and fed him on Carnrick's food, and Carnrick's kumysgen tablets.

He never had any rise in temperature, nor a bad symptom from the time his wounds were dressed till I dismissed him two weeks afterwards. His bowels moved on the sixth day without the slightest pain or inconvenience. He is now working in the field, and is as well as he ever was.

I consider this an extraordinary case, and it goes to prove that if we practice strictly antiseptic precautions and use patience and care in finding and repairing intestinal wounds, the mortality in this class of cases will be greatly lessened.

